# To Do or Not to Do… Primary Health Care Professionals Experiences With Mothers With Children Born of Sexual Violence

**DOI:** 10.3389/fpsyg.2021.708288

**Published:** 2021-09-14

**Authors:** Eline Meuleman, Elisa van Ee

**Affiliations:** ^1^Psychotraumacentrum Zuid Nederland, Reinier van Arkel Groep, ‘s-Hertogenbosch, Netherlands; ^2^Behavioural Science Institute, Radboud University, Nijmegen, Netherlands

**Keywords:** sexual violence, rape born, mother-child relationship, professionals, trauma, primary care

## Abstract

Mothers and their children born of sexual violence are at heightened risk for developing an insecure attachment relationship. These mothers and their children often enter care late or not at all, as they are not identified by health care professionals. In this qualitative study, semi structured interviews were conducted with sixteen professionals in primary care for pregnant women and/or young mothers with the aim to identify the *status quo* in knowledge and skills, challenges, and opportunities. Participants included among others professionals working at Youth Services, psychologists, and clinical nurse specialists. Through a thematic analysis, five themes were identified: the knowledge of the professional, discussing the sexual violence, suitable interventions, points of attention during care, and recommendations. Analysis revealed that three groups of professionals can be distinguished, based on their level of awareness of this target group and their available knowledge and skills. To improve primary care for mothers with children born of sexual violence an increase in awareness, knowledge, and skills is a necessary prerequisite. Scientifically based best practices are therefore necessary for health care professionals to provide adequate care for mothers with children born of sexual violence.

## Introduction

Sexual violence, whether perpetrated by armed forces, strangers or partners, is prevalent worldwide and has far-reaching repercussions for victims. Research reveals the mental, physical, social, and economic harms associated with sexual violence, including anxiety, an elevated risk for chronic diseases, self-blame, and difficulty forming relationships (Armstrong et al., 2018). The connection between sexual violence and mental health is convoluted and bi-directional (Devries et al., 2013). Having a severe mental illness serves as a risk-factor for experiencing sexual violence (Khalifeh et al., 2015). Oppositely, women who have experienced sexual violence have a higher chance to develop and maintain a mental illness (Rees et al., 2011). Breslau et al. (1998) estimated the probability of developing posttraumatic stress disorder (PTSD) after rape as 49%. Rothbaum and Foa (1992) reported that 94% of women experienced PTSD symptoms during the 2 weeks immediately following the rape and about 30% 9 months later Rothbaum et al. (1992). Even though gender-based violence is of all times and ages, the exact prevalence of children born of this sexual violence is still unknown. Human Rights Watch (1996) stated that in Rwanda, from 1994 to 1995, between 2,50,000 and 5,00,000 women were raped. It is estimated that as a consequence of this sexual violence over 1,100 women gave birth to a child, whereas 5,200 chose for an abortion. In the Democratic Republic of Congo the prevalence of sexual violence has been assumed to be 12% among women and the prevalence of sexual violence related pregnancies to be 6–17% among sexual violence survivors (Peterman et al., 2011; Scott et al., 2015). Two years after Islamic State militants attacked the Yazidi religious minority in northern Iraq and took 1,000 of women and girls as sex slaves, many victims returned home pregnant or, in some cases, with newborn babies. According to a report of Amnesty (2020) hundreds of babies were born as a result of sexual enslavement by IS. In 2017, Boko Haram released 82 Chibok schoolgirls in Nigeria. Many of these girls reported to be sexually abused, and some of them came back pregnant or with children. Sexual violence and rape induced pregnancy is a serious contemporary problem that deserves attention.

Research has shown that sexual violence not only affects the wellbeing of victims but also their motherhood. Traumatized mothers are generally less sensitive and less responsive (Schechter et al., 2010; Feeley et al., 2011), less emotional available (van Ee and Kleber, 2013; Cohen and Shulman, 2019) and show more dissociated and insensitive parenting behavior due to memories of the traumatic experience (Green and Goldwyn, 2002). In the case of rape induced pregnancy, a mother has to cope with the social, physical, and mental health consequences of sexual violence while dealing with her pregnancy as a consequence of this violence (Bosmans, 2007; Scott et al., 2015; van Ee and Blokland, 2019). When the child is born, multiple difficulties can arise for the mother while navigating the relationship with her child born of sexual violence. Mothers often connect their traumatic experience to the child, causing some mothers to feel rejection and hatred toward the child, because the child serves as a constant reminder of the sexual violence (van Ee and Kleber, 2013). The child’s identity is likely to be linked to that of their rapist father, reinforced by stigma from the community toward mother and child, even if they never meet the father (van Ee and Kleber, 2013; Scott et al., 2015). These feelings of rejection and hatred toward the child of the perpetrator oscillate with feelings of love for their innocent child. The ambivalence within the mother-child relationship can lead to the formation of an insecure attachment relationship (Bowlby, 1969; van Ee et al., 2016; van Ee and Blokland, 2019).

An insecure attachment relationship may lead to a variety of difficulties in later functioning and even the development of clinical symptoms in the child (Brumariu, 2015). Therefore, the identification of these dyads and the development of a secure attachment should be a focus in early interventions. Prior research has shown that experienced violence in general is under-recognized by mental health professionals (O’Dwyer et al., 2019). Commonly, professionals do not ask about a possible history of violence due to inadequate knowledge, confidence, and skills to respond to such confessions (Rose et al., 2011; Hepworth and McGowan, 2013; Nyame et al., 2013). Moreover, there is no recommended action for mental health services and clinical care to follow up on when a woman has experienced sexual violence (O’Dwyer et al., 2019). In addition, prior research revealed that secondary traumatic stress symptoms are common among professionals who work with traumatized patients (O’Halloran and Linton, 2000; Canfield, 2005). Secondary traumatization (STS) is a result of engaging with clients who tell emotionally shocking material. Pinsley (2000) compared therapists whose caseload consisted above 50% rape or incest survivors with those whose caseloads had fewer than 50%. Results showed that the therapists with high caseloads experienced more symptoms associated with traumatic distress. STS could in turn influence the care provided to mothers with children born of sexual violence. Considering the complexity of the situation of mothers and their children born of sexual violence it is crucial but unknown whether in primary health care these dyads are identified and receive appropriate early interventions.

To our best knowledge, very few studies have included mothers with children born of sexual violence as a target group. Qualitative research showed that experts consider interventions aimed at the mother-child relationship as most effective for mothers with a child born of sexual violence. However, these experts indicated multiple barriers that prevented them from achieving positive outcomes, including a lack of financial resources, a limited evidence base, and treatments starting too late (Anderson and van Ee, 2019). Accordingly, experienced therapists note that mothers with children born of sexual violence often receive care too late, because the connection between the child and the traumatic experience has not been discussed before, even if they are already seen in mental health care (van Ee and Blokland, 2019). Thus, mothers with children born of violence are not identified, or too late, by health care professionals, causing them to not receive the appropriate health care, likely resulting in more difficulties between mother and child and insecure attachment relationships.

There is limited research examining how primary healthcare professionals understand and experience providing care to (upcoming) mothers with children born of sexual violence. This knowledge is integral to provide early high quality care and to prevent insecure attachment relationships between the traumatized mother and child. Therefore, this current qualitative study aims to gain an in-depth understanding of healthcare professionals’ experiences and perceptions in providing primary care for mothers with children born of sexual violence, in order to develop accessible tools for (preventive) care for mothers with a child born of sexual violence.

## Materials and Methods

### Participants

Participants were Dutch healthcare professionals currently or previously working in an organization which provides primary care for pregnant women and/or young mothers. These mothers are mainly immigrants or refugees. We approached multiple organizations throughout the Netherlands *via* email in which it was likely that mothers with children born of sexual violence were seen. Within these organizations, professionals working with (upcoming) mothers were asked to voluntarily participate in this study. Inclusion criteria for professionals were (1) being (or have been) involved in supporting maternity from pregnancy or during the first years of maternity, and (2) being in primary care in the Netherlands. All professionals meeting the inclusion criteria were invited for an online interview to ensure a wide sample was included. No distinction was made between professionals who had a lot of experience with providing care for mothers with children born of sexual violence and those with not much experience. Besides the recruitment *via* email, we used snowball sampling at the end of each interview to find health care professionals in other organizations. We ceased recruitment when no new topics were brought up during the interviews and data saturation was achieved.

### Procedures

Based on a literature review on the needs of mothers with a child born of sexual violence (van Ee and Kleber, 2013) and expert studies (Anderson and van Ee, 2019; van Ee and Blokland, 2019) an interview protocol was developed by a panel of researchers and experienced clinicians and conducted by trained social scientists. The interview contained open-ended questions aimed at constructing a comprehensive picture of the experiences of primary healthcare professionals with providing care for mothers with a (unborn) child from sexual violence. The final guide included questions on: the gathering of information about the background of a mother, barriers to asking questions about sexual violence, factors related to the openness of mothers, best practices, and recommendations for improving the care (see Appendix 1 for the interview guide). Ethical approval was received from the Ethics Committee Faculty of Social Sciences (Radboud University, ECSW-2020-087R1).

Participants were recruited *via* a standardized email sent to organizations throughout the Netherlands. In all organizations, (some) professionals provide care to (upcoming) mothers. Before each interview, participants read the information letter and filled in the informed consent. Semi-structured interviews were, due to the corona crisis, conducted online *via* Microsoft Teams or *via* Zoom. The interview guide was followed and the interviews lasted 60–100 min. In response to questions, participants were encouraged to provide more descriptive details and examples of cases. The interviews were audio-recorded with the participants’ consent and anonymized on transcription.

### Data Analysis

All interviews were transcribed verbatim. To explore the recurrent themes in the data, thematic content analysis was used (Braun and Clarke, 2006). The coding process involved three levels of data coding starting with line-by-line underlining key words and phrases, followed by summarizing key phrases into codes and organizing the data thematically. The analytic process was exploratory in nature. The first two levels of data-coding were carried out by one author to ensure all transcripts were coded consistently. Another member of the research team checked all codes to ensure the key phrases were summarized into the correct code, thereby increasing levels of reliability. The process of summarizing codes into themes was discussed within the research team until consensus on the themes was reached. Open, axial, and selective coding were used to illuminate categories. The coding was extensive and inclusive, and used as the base for this present article. The results for each theme are reported in the next section, and additionally the frequencies of some themes are calculated and presented.

## Results

### Study Participants

Sixteen professionals, all women, were included in this study. The age of the professionals ranged from 30 to 65 years old (*M* = 49). The sample includes four professionals working at Youth Services, three psychologists, three clinical nurse specialists, one midwife, one gynecologist, one social worker, one supervisor of clinical nurse specialists, one parenting supporter, and one parent-child advisor. On average, the professionals have 18 years (*M* = 17.8, SD = 10.6) of experience within their working field.

### Findings

The results are for the purpose of this article divided into five clusters: knowledge of the professional, discussing the sexual violence, interventions, points of attention during care, and recommendations. In Table 1, a quantitative overview of some of the frequently given answers to the open questions in the form of generalized statements can be found. In the next section, these frequently given answers will be elucidated.

**TABLE 1:** Quantitative overview of the results.

Cluster	Frequently given answers on the open-ended questions	Frequency *(N)*
Knowledge	My knowledge is based on my previous experience with the target group	10
	I (partly) miss knowledge needed to provide adequate care	6
	I (partly) miss knowledge about different cultures, needed to provide adequate care	6
Discussing sexual violence	It’s important to discuss possible experienced sexual violence with mothers	16
	I don’t have struggles with interrogating about sexual violence	9
	I regularly ask mothers about possible experienced sexual violence	9
	I have struggles with interrogating about sexual violence	7
	It’s important to guide the boundaries of the mother when discussing the sexual violence	7
	I don’t ask mothers about possible experienced sexual violence	6
	I standardly ask mothers about possible experienced sexual violence in the intake conversation	6
	It’s important to explain mothers why you ask about sexual violence	3
Interventions	Mothers are willing to improve the mother-child relationship	16
	I provide mother-child interaction therapy	13
	I provide interventions targeting the mother	11
	I provide psychoeducation to the mother	9
	I involve the system of the mother in her treatment or care	4
Points of attention	A trusting therapeutic relationship is important	13
	At first, most mothers are not willing to accept help	12
	Most mothers feel too ashamed to talk about sexual violence	11
	I have been negatively affected by the maternal trauma. (secondary traumatization)	10
	I can easily reach out for external help	10
	I motivate mothers to seek external assistance, when I can’t help them myself	8
	Customized care provided to these mothers is key	7
	The waiting times for the referral of mothers to another organization is too long	6
	Early identification of these mothers is key	6
	Most mothers do instantly cooperate with the help provided	4
Recommendations	I wish for more supervision and intervision	14
	I wish for increasing my knowledge about sexual violence	9
	I wish for improving my skills when it comes to providing care to mothers with children born of sexual violence	9
	I recommend standardly asking mothers about possible sexual violence in the intake conversation	5
	I want to know better where to refer a mother to	3

#### Knowledge

The first cluster of results focuses on available knowledge of the professional, covering both general knowledge about sexual violence and knowledge about different cultures. Six professionals mentioned they (partly) miss the needed knowledge to provide adequate care for mothers with a child from sexual violence. Professionals stated they are unbeknownst about the re-experience of the traumatic violence, suitable conversational skills, and the impact of sexual violence on motherhood. One professional, a supervisor of clinical nurse specialists with over 12 years of experience, elucidated that this lack of knowledge exists as a consequence of the low prevalence of mothers with children born of sexual violence.

Many care providers see these mothers so little. Of course, it is not something that occurs once in every 10 women. So sometimes it is such a coincidence that you can hardly blame that they are not well trained or educated.

Insights from our analysis show that professionals working in social services, obstetrics, or nursing, often have insufficient knowledge about this relatively small group of mothers. On the other hand, professionals expand their knowledge once they become more experienced in providing care to mothers with a child from sexual violence. Ten professionals pointed out that their available knowledge is based on their previous experience with the target group. A professional working in Youth Services, with 15 years of experience, acknowledged her own previous lack of skill. She did substantially improve her skills by gaining more experience, but is still unsure about her competence.

I am not really prepared to work with mothers with a child from sexual violence. I gradually gained experience. The hardest part is, that if your knowledge is not sufficient, you don’t really know what you’re missing. I think I do miss some knowledge.

An interesting question related to this topic is whether professionals require some knowledge about mothers with children born of sexual violence, e.g., common risk-factors or signals, to be able to recognize and identify them. As one participant illustrated, nurses have to become aware of the possibility that sexual violence might have caused a pregnancy and inquire, because most mothers won’t speak up by themselves. Consequently, if they do, professionals gain more knowledge about mothers with a child born of sexual violence and its consequences. More knowledge can in turn lead to more awareness and a higher likelihood that a professional will recognize and identify this target group.

In addition to having knowledge about sexual violence, it’s important for some professionals to have knowledge about different cultures. Six professionals mentioned that they are not familiar with different cultures, which complicates the care they provide. These professionals emphasized that too many cultures exist, with too many specific characteristics to learn by heart. Nevertheless, they do endorse the importance of multiculturalism, because of the impact culture diversity can have on the efficacy of interventions.

#### Discussing Sexual Violence

All participants touched on the dilemma whether or not to discuss the (potential) sexual violence with pregnant women or mothers. In this paragraph, we differentiate between explicitly asking the mother about the (potential) sexual violence and further investigating the sexual violence and its consequences.

First of all, six professionals reported that they don’t explicitly ask a mother about the circumstances around her pregnancy. The main reasons why these professionals refrain from discussing this topic are: not being aware that the mother might have experienced sexual violence and became pregnant as a consequence, not knowing how to help the mother after she has told her story, not knowing which words to use to address the topic, feeling like the topic is too heavy, and having to ask many questions in the intake and therefore not being able to discuss all topics. However, all professionals mentioned the importance of discussing possible sexual violence, because of the impact of the traumatic experience on the mother-child attachment relationship. Three participants brought up the importance of explaining to the mother why they ask her about (potential) sexual violence. One clinical psychologist with 25 years of experience told she usually explains a mother how maternal traumatic experiences impact the child, to make sure she understands the importance of telling her story.

Yes, it is sometimes tensive to ask it, but I have also learned that it provides a lot of information. I always explain well why I ask that question, why it is important. Sometimes you see a mother is looking like ‘why are you asking me this?’ So then you delve into it, to explain why it is important and then you also explain that tensions or stress in pregnancy can affect the unborn child. I will always ask about it. But you really have to ask, they are not going to say it on their own.

Nine professionals reported they do regularly ask about pregnancy wishes and circumstances. Reasons why they discuss the sexual violence include adjusting the course of treatment or care (e.g., a slower course, providing customized care), offering trauma treatment, enhancing the safe attachment relationship between mother and child, and/or improving childbirth.

Signals used by the professionals to recognize that a mother might have experienced sexual violence, and became pregnant as a consequence of it, are: a disorganized attachment relationship, hostile communication between father and mother (if the father is in the picture), body language of the mother (e.g., not being happy with her own body), and negative thoughts of the mother surrounding the (unborn) child. Six professionals stated that they standardly ask questions related to sexual violence in the intake conversation. However, not all mothers are ready to tell their whole story during the intake. One nurse, with 10 years of working experience, pointed out that during intakes questions about sexual violence are routinely asked, but not all mothers are ready to tell their story during the first meeting. Making sexual violence negotiable during the treatment trajectory remains key.

Well, of course we do an extensive intake in which we also indicate that we have quite intensive questions for them, including whether they have experienced sexual violence themselves and whether that is still going on at the moment. But of course, mothers do not have to tell everything during an intake, so sometimes you come across things during the 2.5 years that you are involved with them.

Therefore, it’s important that a professional is able to further inquire about sexual violence. Seven professionals talked about their own struggle with interrogating. The main explanation for this struggle is the fear of causing harm to the mother when bringing up the traumatic experience and not being able to help the mother afterward. As one nurse explained, the fear of being damaging rather than helpful refrains her from asking a mother about sexual violence.

To what extent do I break something open again or do I cause more damage. What results do I get following my question? Can I do something with the answers? Maybe I get an answer of which I think “oh shit what should I do next, because it is beyond my expertise.”

Moreover, some professionals revealed shyness prevented them from discussing sensitive topics. Not finding the right words to address the sexual violence plays a role for some professionals, causing them to not inquire further.

On the other hand, nine professionals do not face (huge) difficulties to inquire about the sexual violence with the mother. Almost all of these professionals mentioned that due to their gained experience, they advanced in asking questions about sexual violence. Their coping strategies include controlling their own emotions when a mother tells about her traumatic experiences, knowing when to start and stop interrogating based on the reactions and body language of the mother, using the right words to encourage mothers to speak up, making the mother feel at ease before interrogating, explaining why discussing certain topics is important for both mother and child, and feeling competent to help the mother after she has told her story (e.g., knowing where to refer her to or which therapy to apply).

Last of all, seven professionals spoke of the importance of guarding the personal boundaries of the mother when discussing the sexual violence. Keeping the pace of the client is crucial. While working with this target group, professionals declared that a wait-and-see attitude needs to be adopted. The supervisor of clinical nurse specialists explained that most mothers’ boundaries have not only been crossed, but violated, which constitutes exploitation. In order to establish a good therapeutic or professional relationship, guarding the mothers’ boundaries is crucial.

I learned very early on in my career that it’s important to guard their boundaries. Their boundaries got violated in the past and care providers are not allowed to violate those boundaries again. So, if the time isn’t right to talk about it, that’s fine too. It can also be done later.

#### Interventions

The professionals use different interventions focusing mainly on the mother-child relationship, the mother, education and/or the system. The intervention provided depends on the employment of the professional. Thirteen professionals provide mother-child interaction therapy, for example an attachment-based parenting intervention called NIKA or Video Home Training. Mother-child interaction therapies are often given in addition to trauma therapy focusing solely on the mother. Professionals mentioned that the attachment relationship between mother and child is often distorted due to traumatic experiences. Based on their clinical expertise, professionals pointed out that mothers link certain traits or behavior of their child to the perpetrator, which causes them to re-experience the trauma. However, all professionals portrayed the mothers as being willing to strengthen the mother-child relationship.

Eleven professionals provide interventions targeting only the mother, ranging from lending a sympathetic ear to offering eye movement desensitization and reprocessing (EMDR)-therapy. All professionals confirm the importance of offering trauma treatment to the mother, because of the negative influence of maternal traumas on the mother-child attachment relationship. Psychoeducation is also commonly applied by the professionals. Nine professionals highlighted that they clarify the effects of traumatic experiences on the relationship with her child, without blaming her for the sexual violence. Last of all, four professionals mentioned the importance of involving the system around the dyad in the care or treatment (e.g., involving other family members).

#### Points of Attention

The professionals named five points of attention when it comes to providing care to mothers with children born of sexual violence: the therapeutic relationship, cooperation of mothers, empathic engagement, external help and referral, and attitude changes.

First of all, 13 professionals introduced the importance of a trusting therapeutic relationship. Accordingly, mothers need to feel safe before disclosing their personal stories. Professionals try to create a pleasant atmosphere, by adopting an inviting attitude and making the mothers feel at ease. Contrarily, one professional noted that though she wants to be reliable, she thinks it’s not necessary to build up relationships with all mothers. She described the relationship with the mothers more as a trade-off: she has something to offer (help) and the mothers have to choose whether they want the help. This opinion is the exact opposite of the other opinions. Most professionals, like a psychologist with 25 years of work experience, stressed shared trust is needed before a mother opens up.

First you have to invest much in the relationship of trust, before mothers even dare to talk about the sexual violence. They are people who have become so damaged in their trust in fellow human beings; they won’t just show the back of their tongue. It is not that they don’t want to, but it’s their survival strategy. If a mother can’t trust anyone, why would she tell me everything. It really takes time.

Secondly, all professionals talked about the degree to which mothers cooperated with the available care. Twelve professionals stated that most mothers with children from sexual violence were (at least firstly) not willing to accept the support provided by the professional, whereas four professionals declared that most mothers did cooperate. A psychologist, who has 35 years of working experience, explained that the main reason why mothers don’t open up for the available help is their avoidance toward the traumatic experience.

In many cases, being open to assistance after experiencing sexual violence is something that people do not really like to do. One of the characteristics of PTSD is that you want to avoid the trauma, that you don’t want to think about it, that you don’t want to be involved with it. It’s one of the most difficult topics to ask help for.

Besides, eleven professionals indicated that some mothers feel too ashamed to talk about the sexual violence, leading to reluctance toward any form of support. Different factors influence the degree to which mothers feel ashamed, including the culture of the mother, fear for reactions of family or friends, taboos around both sexual violence and seeking help when pregnant, blaming oneself for the sexual violence and having little trust in professionals.

Aside from avoidance and shame, other reasons mentioned by the professionals as to why mothers don’t cooperate are: fear of losing the child, thinking sexual violence is normal, needing only help for the child and not for themselves or needing no help at all, secrecy around the sexual violence (not wanting the family to find out about it), and being too mentally damaged. The main reason why mothers do eventually open up for the support provided by the professionals is the love for their child.

Thirdly, 10 professionals feel like they have been negatively affected by the maternal trauma, through empathic engagement with the mother. Professionals have to be able to deal with horrible stories and have to endure the behavior of the mother toward the professional and child. Prevention of STS is important, as noted by one professional with a supervising role.

Hearing stories of very intense things all day… you have to deal with that. If you do not detoxify enough of those stories then the chance of STS is certainly present. You can become sick of the stories you hear.

A fourth point of attention relates to the ease in which professionals can reach out for external help. Ten professionals named their organization works together closely with other organizations specialized in for example trauma treatment, parent-child support, youth protection, maternity care or nursing. This collaboration makes a referral to another provider, which is often needed due to the complexity, more easy. Four professionals can refer to or consult with a psychologist, two have sessions of intervention with colleagues and three benefit from supervision within their organization.

On the other hand, six professionals complained about the long waiting times before a mother gets help when she is referred to another organization. Moreover, one professional disapproved that information about the background of a mother is not shared between organizations due to privacy concerns. According to this professional, the lack of sharing complicates the provision of care. Eight professionals talked about their efforts to motivate mothers to seek external assistance, for example to visit a psychologist. The professionals motivate mothers by arranging new external assistance, accompanying mothers during their first intake conversation, making clear mothers can always return for help and staying involved with the mothers.

Last of all, professionals mentioned how they adjust their attitude toward the mother, when they know the mother experienced sexual violence. Most commonly, professionals adopt a cautious, respectful and empathetic attitude, and weigh their words more carefully. Six professionals discussed the importance of early identification of these mothers and starting quick with the intervention. Moreover, mothers have to be encouraged to take the lead in determining the speed of treatment. In addition, seven professionals considered customized care of great importance, meaning that the treatment plan has to be flexible.

#### Recommendations

The professionals recommended that different types of assistance are needed for themselves to improve their care provided to mothers with children born of sexual violence, including taking part in supervision and intervision, increasing knowledge, improving skills, knowing how to provide best care, and using standardized questions in the intake conversation. Fourteen professionals wished for good sessions of supervision and intervision. They asked for an expert supervisor who keeps an eye on both mother and professional, and intervision groups to be able to ask for advice and share stories about clients with other professionals.

Secondly, nine professionals aspire to increase their knowledge about sexual violence. One mayor topic includes the effects of sexual violence on the upbringings of a child. Moreover, professionals noted the importance of obtaining knowledge about signals, risk-factors, and trauma triggers, to be better able to signal and help mothers with children born of sexual violence. In addition, professionals want to obtain more knowledge about different cultures and their influence on treatment and mothers themselves.

Thirdly, nine professionals stated they feel the urge to improve their skills. Most commonly, professionals want to enhance their conversational skills, including discussing sexual violence and identifying mothers who experienced sexual violence through asking specific questions. Professionals mentioned the need for sample questions and practice sessions, giving them tools to handle and give direction to conversations in which sexual violence is discussed. Professionals want to adjust their attitude to the target group, e.g., by not avoiding difficult situations, asking all questions, and maintaining the boundaries of the mothers. Moreover, understanding signals, identification of mothers who have children from sexual violence, and the ability to handle the conversation are seen as important skills which are often underdeveloped.

Fourthly, professionals said they want to learn more and possess more skills about how to provide the best care possible for mothers with children born of sexual violence. Professionals worry they can’t help the mother after she has told her story. Therefore, three professionals declared they want to make external assistance more visible by knowing better where to refer a mother with children from sexual violence to. On the other hand, some interviewed professionals provide treatment themselves. Four of them, including a psychologist and sexuality consultant, desire to provide specific treatment methods including EMDR, cognitive behavior therapy, and Narrative Exposure Therapy (NET). Two professionals noted the importance of a multidisciplinary approach when treating mothers with a child from sexual abuse, including one psychologist with over 30 years of experience. She stated that therapists need to apply diverse interventions to sufficiently help mothers with children born of sexual violence.

I would recommend that a therapist has a broader scope, so you can treat different kinds of trauma or you can integrate methods. For example, a therapist can’t solely offer EMDR. I wouldn’t recommend that; a protocolized treatment. It is simply too complex for that.

Last of all, five professionals recommended standardly asking pregnant women or mothers about possible sexual violence in the intake conversation. One professional proposed the use of priming within (all kinds of) intake conversations. The prime consists of sentences in which the professional acknowledges that not all children are born out of pleasant circumstances, which makes women with children from sexual violence more likely to speak up. By not always assuming that a mother is happy with her child, mothers with children from sexual violence might feel more heard and safer to tell their story.

## Discussion

### Conclusion and Discussion

The present study identified the perceptions and experiences of 16 professionals with providing primary care to mothers with children born of sexual violence. Mothers with children born of sexual violence, often immigrant or refugee, regularly experience difficulties in regulating the attachment-relationship with their child. An insecure relationship may lead to negative prospects for the child, including difficulties in later functioning and the development of clinical symptoms (Brumariu, 2015). Though the negative impact of sexual violence on both mother and child, the exact number of children conceived from an act of sexual violence remains unknown (van Ee and Blokland, 2019). Moreover, prior research revealed mothers and children often come into care late, because they are not signalized by health care professionals (van Ee and Blokland, 2019). Therefore, improving primary care provided to this target group and thereby preventing the development of an insecure relationship between mother and child is crucial. Indeed, all professionals in the present study mentioned the importance of a safe and stable mother-child attachment relationship.

Our findings suggest that primary health care professionals can be divided into three groups: those who were not aware of the possibility that a mother has become pregnant because of sexual violence; those who recognized the prevalence of sexual violence but felt unequipped to ask appropriately and provide the help necessary; and those who understood the importance of discussing the sexual violence and acted accordingly. Our analysis showed that professionals working in social services, obstetrics or nursing, often have insufficient knowledge about mothers with children born of sexual violence. These professionals therefore have a higher chance to belong to the first or second group. Moreover, as described in our findings, the more experienced the professionals become, the better care they are able to provide. Hence, professionals with more years of experience within their working field are more likely to belong to the third group. Several steps need to be taken in order to minimize the amount of health professionals belonging to the first or second group, such as giving knowledge-training with experiential exercises in which culture-sensitive conversational skills are practiced to minimize those professionals who are unaware of the target group, and setting up intervision groups and consultation to make professionals more equipped. Minimizing the first and second group will probably lead to a faster identification of mothers with children born of sexual violence.

Herein, our findings echo several results from studies focusing on the experiences of health care professionals with providing care to women who experienced violence. O’Dwyer et al. (2019) also found that a lack of experience leads to more feelings of fear and inadequacy. Moreover, mental health professionals likewise don’t routinely ask about childhood sexual abuse during assessments (Hepworth and McGowan, 2013). In addition, the interviewed professionals also experience (symptoms) of STS, which are found to be common for professionals working with traumatized patients (O’Halloran and Linton, 2000; Pinsley, 2000; Canfield, 2005). Prior research also identified recommendations to improve healthcare services, among those there are recommendations for training of professionals to increase their confidence and expertise (Rose et al., 2011) and recommendations for clear referral pathways (Nyame et al., 2013). This study contributed to the literature by showing the challenges of health care professionals associated with providing care to mothers with a child born of sexual violence. In addition, it resulted in the development of a website for mothers, their children born of sexual violence as well as professionals to increase access to knowledge, skill training, intervention, and consultation.

### Limitations

All interviews have been carried out within organizations that voluntarily participated after receiving an e-mail. Assuming that organizations and health care professionals are more prone to volunteer when they are aware of the target group and have a somewhat positive self-image, this may suggest that the sample is positively skewed. Yet, two health care professionals who had never (consciously) provided care to mothers with children born of sexual violence did participate.

Secondly, as with all interview studies, there might be a discrepancy between what health professionals say and/or perceive and the actual practice. Many questions were asked about inquiring about possible sexual violence, but observations were not part of this study. Therefore, no claims can be made about the actual practice of health care professionals in this sample.

A third limitation concerns the location of the study. While most mothers with a child born of sexual violence reside in (former) war zones, this study was performed within the Netherlands among professionals working with refugees, migrants or Dutch victims of sexual violence. Health care and the needs of these mothers and their professionals, as well as related transcultural aspects, may differ when provided in a Western country or a (former) war zone.

A final limitation relates to the thematic analysis performed. Statements made by participants can be categorized in multiple ways. Therefore, the identified themes are possibly not exclusive nor exhaustive, as is often the case with a thematic analysis. Since only one author coded the transcripts and the study was exploratory in nature, this study does not include a measurement for inter-coder reliability.

### Implications for Future Research

In this study, included participants were primary health care professionals (possibly) providing care to mothers with children born of sexual violence. The findings from this research can be extended in two different ways.

First of all, the findings can be used as a base for the design of a survey. This qualitative study has included a limited number of organizations and health care professionals, which makes the generalization of the findings difficult. Similar questions can be investigated quantitatively within a larger sample of participants by conducting a survey. Since the prevalence of mothers with a child born of sexual violence is low, this research area would benefit from a survey distributed to a large sample of health care professionals working with (upcoming) mothers.

Secondly, the health care professionals mentioned their difficulties with for example choosing the right words to address possible sexual violence and knowing when to start and stop inquiring about sexual violence. This research domain would benefit from conducting interviews with these mothers to understand their experiences, preferences, and possible pitfalls of health care professionals.

## Data Availability Statement

The raw data supporting the conclusions of this article will be made available by the authors, without undue reservation.

## Ethics Statement

The studies involving human participants were reviewed and approved by the Ethics Committee Faculty of Social Sciences. The patients/participants provided their written informed consent to participate in this study.

## Author Contributions

EE contributed to the conception and design of the study, and wrote the sections of the manuscript. EE and EM organized the database. EM performed the interviews and analysis, and wrote the first draft of the manuscript. Both authors contributed to the manuscript revision, read, and approved the submitted version.

## Conflict of Interest

The authors declare that the research was conducted in the absence of any commercial or financial relationships that could be construed as a potential conflict of interest.

## Publisher’s Note

All claims expressed in this article are solely those of the authors and do not necessarily represent those of their affiliated organizations, or those of the publisher, the editors and the reviewers. Any product that may be evaluated in this article, or claim that may be made by its manufacturer, is not guaranteed or endorsed by the publisher.

## Appendix 1. Interview Guide

(1) As a professional, you guide women during/after pregnancy in motherhood. Are you aware of any mothers who have experienced sexual violence?
(2) How often has it happened, in your practice, that a woman was pregnant or had a child due to sexual violence?
(3) How did you get information about the circumstances around the pregnancy of a woman? (The mother mentioned it herself/You were informed by the referrer/You asked about a history of sexual violence/Otherwise, namely…)
(4) If you asked about the sexual violence yourself, what were signals that prompted you to inquire about the desirability and conception of the child?
(5a) Are you experiencing obstacles that prevent you from asking questions about sexual violence? If yes, which obstacles?
(5b) Do you recognize the examples of obstacles below?
  – obstruction due to the attitude of the mother/the contact you have with her?
  – obstruction because you think it might not be culturally sensitive to ask these questions?
  – obstruction because you feel that it is not part of your duties to ask this question?

(6) Has it ever happened that you did not want or dare to ask questions related to sexual violence, when you suggested the mother had a child born of sexual violence?
(7) Do you include knowledge about mothers with a child born of sexual violence in the guidance you provide? If so, how?
(8) What needs for help or support did you identify in these mothers and children?
(9) Were the mothers you knew open for help?
(10) Which factors do you think play a role if mothers do not want to accept help (e.g., shame, ambivalence, language problems, cultural differences, ignorance)?
(11) How do you try to deal with these difficulties and obstacles?
(12) Can you give examples of help that you offer?
(13) Was it easy to refer to another organization? Can you provide an explanation for both yes and no answers?
(14) What assistance would you consider most appropriate for this target group (whether provided by you, your organization, or another organization)?
(15) Can you explain why you would find this help most appropriate? What are your considerations?
(16) What help would you not recommend?
(17) Do you feel like you lack information, knowledge or skills to provide good care for these mothers?
(18) What need for support do you, as a care provider, have when working with these problems?
(19) What information do you think could be important to provide through a website?
(20) What self-help tools do you think would be helpful?
(21) Finally, has there been a mother, where sexual violence played a role, who you still remember clearly? Why did this case stay with you? What could have helped you to provide better care.
